# The effect of artemisinin-combination treatment options on *P. falciparum* gametocyte carriage: a pooled analysis of individual patient data

**DOI:** 10.1186/1475-2875-13-S1-P44

**Published:** 2014-09-22

**Authors:** Georgina Humphreys

**Affiliations:** 1 WorldWide Antimalarial Resistance Network (WWARN), Oxford, UK; 2 Centre for Trop. Med., Nuffield Dept. of Clin. Med., Univeristy of Oxford, Oxford, UK

## Background

Artemisinin combination therapies (ACT) rapidly clear the asexual parasite population in infected individuals. In addition, the artemisinin components specifically kill early stages of gametocyte development and have an incomplete effect against mature gametocytes. These properties mean that ACT may play a critical role in reducing the transmission of malaria and decreasing the spread of drug resistant parasites. However, different ACT options may have differential effects on gametocyte carriage and the production of gametocytes during infections is influenced by parasite, human and environmental factors.

## Material and methods

A systematic search of the literature was conducted to identify all studies published between 1960 and March 2014, in which patients were enrolled and treated with antimalarials and where gametocyte data were recorded. An a priori data analysis plan was developed to identify factors associated with gametocyte prevalence and density prior to treatment and following treatment with ACT. Individual patient data from over 100 published studies (n > 50,000) were collated, curated and included in the analysis, with data from 21 African and 6 Asian countries. Criteria for the quality of gametocyte assessments were ascribed to the various studies.

## Results

Before September 2014, we will have determined the effects of asexual parasite density, age, transmission intensity and haemoglobin concentration on enrolment gametocyte prevalence and density. We will further present post-treatment gametocyte prevalence, density and carriage time in relation to ACT regimen (artemether-lumefantrine, amodiaquine-artesunate, dihydroartemisinin-piperaquine, and mefloquine artesunate), parasite clearance time, transmission intensity and human host factors.

